# Oridonin ameliorates renal fibrosis in diabetic nephropathy by inhibiting the Wnt/β-catenin signaling pathway

**DOI:** 10.1080/0886022X.2024.2347462

**Published:** 2024-06-04

**Authors:** Jushuang Li, Lan Shu, Qianqian Jiang, Baohong Feng, Zhimin Bi, Geli Zhu, Yanxia Zhang, Xiangyou Li, Jun Wu

**Affiliations:** a Department of Nephrology, Tongren Hospital of Wuhan University (Wuhan Third Hospital), Wuhan University, Wuhan, P.R. China; b Network & Informatization Office, Huazhong University of Science and Technology Hospital, Wuhan, P.R. China; c Department of Nephrology, Wuchang Hospital, Wuhan University of Science and Technology, Wuhan, P.R. China

**Keywords:** Oridonin, diabetic nephropathy, renal fibrosis, Wnt/β-catenin

## Abstract

Diabetic nephropathy (DN) is one of the most serious and frequent complications among diabetes patients and presently constitutes vast the cases of end-stage renal disease worldwide. Tubulointerstitial fibrosis is a crucial factor related to the occurrence and progression of DN. Oridonin (Ori) is a diterpenoid derived from *rubescens* that has diverse pharmacological properties. Our previous study showed that Ori can protect against DN by decreasing the inflammatory response. However, whether Ori can alleviate renal fibrosis in DN remains unknown. Here, we investigated the mechanism through which Ori affects the Wnt/β-catenin signaling pathway in diabetic rats and human proximal tubular epithelial cells (HK-2) exposed to high glucose (HG) levels. Our results revealed that Ori treatment markedly decreased urinary protein excretion levels, improved renal function and alleviated renal fibrosis in diabetic rats. *In vitro*, HG treatment increased the migration of HK-2 cells while reducing their viability and proliferation rate, and treatment with Ori reversed these changes. Additionally, the knockdown of β-catenin arrested cell migration and reduced the expression levels of Wnt/β-catenin signaling-related molecules (Wnt4, p-GSK3β and β-catenin) and fibrosis-related molecules (α-smooth muscle actin, collagen I and fibronectin), and Ori treatment exerted an effect similar to that observed after the knockdown of β-catenin. Furthermore, the combination of Ori treatment and β-catenin downregulation exerted more pronounced biological effects than treatment alone. These findings may provide the first line of evidence showing that Ori alleviates fibrosis in DN by inhibiting the Wnt/β-catenin signaling pathway and thereby reveal a novel therapeutic avenue for treating tubulointerstitial fibrosis.

## Introduction

At present, the global incidence of diabetes is rapidly increasing, and because there are few treatment alternatives for the prevention of diabetic nephropathy (DN), the incidence of DN is also expected to increase [1,2]. DN is currently the leading cause of end-stage renal disease (ESRD) worldwide [3,4]. However, the pathogenesis of DN is very complex and has not been elucidated. Tubulointerstitial fibrosis is a major contributor to the onset and development of DN [5,6]. Tubular epithelial cells (TECs), which constitute a significant portion of the renal parenchyma and are susceptible to destruction during kidney injury, are responsible for the development of kidney fibrosis and progressive chronic kidney disease (CKD).

Early studies suggested that the primary site of damage in DN was the glomerulus, however, as our understanding of the pathogenesis of this disease has advanced, it has become clear that there is also involvement of the renal tubules. Tubular abnormalities develop independently of glomerular changes and have a more important impact on renal function than do glomerular alterations, thereby changing the previous viewpoint that was centered on the glomerulus [7,8]. Tubular structural and functional changes precede glomerular damage in high-sugar states, increased glucose reabsorption and secretion by tubular cells lead to tubular hypertrophy, which which is one of the earliest manifestations of DN [9]. Tubulointerstitial fibrosis is the common pathway and main pathological basis for the progression of various chronic kidney diseases, including DN, to end-stage renal failure [10]. Tubular damage and interstitial fibrosis are increasingly being recognized for their roles in the pathogenesis and progression of DN by numerous researchers.

The Wnt/β-catenin signaling pathway is closely related to the occurrence and progression of kidney disease. Numerous studies have shown that the activated Wnt/β-catenin signaling pathway plays a crucial role in promoting renal fibrosis, primarily by controlling the expression of various downstream mediators associated with renal fibrosis [11–13]. In an ischaemia–reperfusion injury (IRI) model, high expression of indoleamine-2,3-dioxygenase (IDO) was shown to activate the Wnt/β-catenin pathway, leading to renal fibrosis. In addition, by inhibiting IDO expression and reducing β-catenin expression, prostaglandin E2 (PGE2) can downregulate the expression of α-smooth muscle actin (α-SMA) and fibronectin to ameliorate renal fibrosis [14]. Zhou et al. reported that overexpression of cannabinoid receptor type 2 promotes kidney fibrosis through orchestrating β-catenin signaling both *in vivo* and *in vitro* [15]. In summary, sustained activation of Wnt/β-catenin signaling is a powerful promoter of renal fibrosis. Therefore, inhibiting the Wnt/β-catenin signaling pathway could be a potential promising therapeutic target for alleviating renal fibrosis.

**Figure 1.**
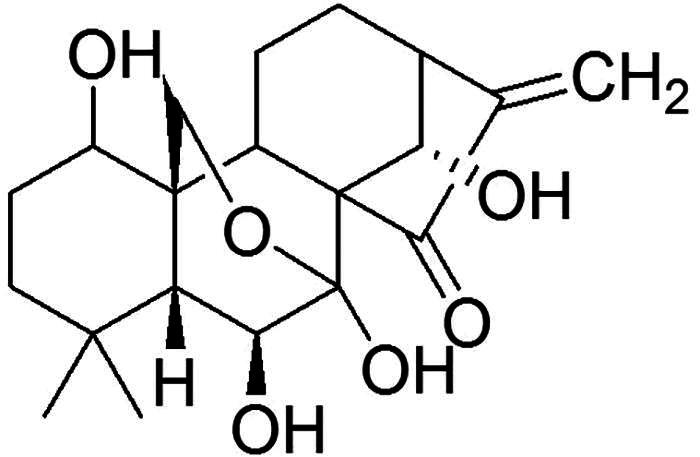
Chemical structure of oridonin.

Oridonin (Ori, Figure 1) is a diterpenoid derived from *Rabdosia rubescens* that has a wide range of pharmacological effects, including antifibrotic, antitumour, anti-inflammatory, immunoregulatory and antioxidant effects [16–21]. Yang et al. reported that Ori alleviated lipopolysaccharide (LPS)-induced early pulmonary fibrosis by restraining autophagy, oxidative stress, NOD-like receptor thermal protein domain associated protein 3 (NLRP3)-dependent inflammation and epithelial mesenchymal transformation [22]. Several studies have also shown that Ori can reduce mild myocardial fibrosis and liver fibrosis by inhibiting the NLRP3 inflammasome [23,24]. Our previous study revealed that Ori exerts a protective effect on DN by blocking the Toll-like receptor 4 (TLR4)/p38-mitogen-activated protein kinase (MAPK) and TLR4/nuclear factor-κB (NF-κB) signaling pathways [25]. In this study, we sought to determine whether Ori reduces renal fibrosis in diabetic rat models and human TECs exposed to high glucose (HG) levels by blocking the Wnt/β-catenin pathway. This research might offer a molecular foundation for a new therapeutic target in DN.

## Materials and methods

### Materials

Human TECs (HK-2) were purchased from the China Center for Type Culture Collection (CCTCC, Wuhan, China). Oridonin (Ori) was obtained from Shanghai Yuanye Biological Technology Co., Ltd. (Shanghai, China), and its purity (over 98%) was determined *via* high-performance liquid chromatography (HPLC). Streptozotocin (STZ) was obtained from Sigma (St. Louis, MO, USA). The kits used for the blood urea nitrogen (BUN) and serum creatinine (Scr) assays were purchased from Changchun Huili Biotech Co., Ltd. (Changchun, China), and the kits for the urine protein assay were obtained from the Nanjing Jiancheng Institute of Biotechnology (Nanjing, China). The cell Counting Kit-8 (CCK-8) was used for this study. Antibodies against collagen I (Col I) were obtained from Abcam (Cambridge, England), and fibronectin (FN) was obtained from Proteintech Group, Inc. (Wuhan, China). SYBR® Premix Ex Taq™ (Vazyme, Nanjing, China) was used for the quantitative polymerase chain reaction (qPCR) assay. Predesigned small interfering RNAs (siRNAs) against rat β-catenin and the control scrambled siRNA were obtained from RiboBio Co., Ltd. (Guangzhou, China).

### Animal models

The animal model of DN was established according to our previous report [24]. Briefly, 24 male Sprague–Dawley rats without specific pathogens and weighing 180–220 g were obtained from the Animal Research Center of Wuhan University. During the whole experiment, the rats were maintained in standard laboratory settings with access to food and water without restriction, a temperature of 21 ± 2 °C, a humidity level of 55 ± 2%, and 12-h cycles of light and darkness. A total of 24 rats were randomly assigned to the normal control (NC) group (*n* = 8), the DN group (*n* = 8), or the DN + Ori group (*n* = 8). After four weeks of feeding a high-fat diet (D12492, Jiangsu Xietong Pharmaceutical Bioengineering Co., Ltd.), the rats in the DN and DN + Ori groups were administered an intraperitoneal injection of STZ at a dose of 35 mg/kg, which was diluted in 0.1 mol/L citric acid buffer (pH 4.3). After being fed the standard diet for 4 weeks, the rats in the NC group received an intraperitoneal injection of the same dosage of citrate buffer (0.1 mol/L, pH 4.3). To evaluate the random blood glucose levels of the rats, blood samples were obtained from the tail vein 72 h after STZ injection. A random blood glucose level greater than 16.7 mmol/L indicated that the diabetes model was successfully established. Afterwards, the rats in the DN + Ori group received an intraperitoneal injection of Ori (10 mg/kg) [25,26] every morning for 10 weeks, whereas those in the NC and DN groups received daily intraperitoneal injections of an equal volume of saline. The random blood glucose levels and body weights of all the rats were monitored weekly. At the end of the 10th week, the rats were maintained in metabolic cases, given *ad libitum* access to feed and water for 24-h urine collection and then killed under 5% chloral hydrate anesthesia. Blood samples were collected by heart puncture. The serum was then separated by centrifugation and stored at −20 °C for future assays. In addition, kidney tissues were removed, frozen in liquid nitrogen and stored. The Animal Ethics Committee of Tongren Hospital Affiliated with Wuhan University (Wuhan, China) approved the experimental protocols for all the animals.

### Assessment of renal biochemical indicators

The 24-h urine protein concentrations and the Scr and BUN levels were determined by using relevant assay kits according to the manufacturer’s instructions.

### Histopathological analysis of kidneys

Rat kidney tissues were fixed with 4% paraformaldehyde and embedded in paraffin, and the embedded tissues were sliced into 4-μm sections. The sections were mounted on clean glass slides, deparaffinized and subjected to periodic acid-Schiff (PAS) and Masson’s trichrome (MT) staining. Images were obtained under a light microscope (Olympus, Japan).

### Immunohistochemistry

For immunohistochemistry, paraffin-embedded kidney tissue sections were deparaffinized and dehydrated according to a standardized method. After antigen retrieval, the sections were blocked with 5% fetal bovine serum (FBS) blocking solution for 30 min at room temperature and then incubated with diluted primary antibodies against Col I and FN at 4 °C overnight. The slices were subsequently washed with phosphate-buffered saline (PBS) and then incubated for 30 min with secondary antibodies at 37 °C. Freshly prepared diaminobenzidine (DAB) solution was added to each section. The tissue samples were analyzed using a light microscope. The immunohistochemical staining images were quantitatively analyzed using Image-Pro Plus 6.0 software (Media Cybernetics Co., Ltd., USA).

### Cell culture and drug administration

HK-2 cells were incubated in Dulbecco’s modified Eagle’s medium (DMEM)/low glucose plus 10% FBS at 37 °C in a humid environment with 5% CO_2_. After being subjected to serum starvation for 24 h at subconfluence, the cells were treated with high glucose (HG) (30 mM) with or without Ori (5 μmol/L) according to our previous report [25].

### Cell transfection

β-Catenin overexpression vectors and corresponding empty vectors (pcDNA3.1) were purchased from GenePharma. HK-2 cells were transfected with siRNAs against β-catenin and β-catenin overexpression vectors using Lipofectamine 2000 (Invitrogen, Carlsbad, CA, USA), and the expression of β-catenin mRNA was assessed *via* qPCR.

### Cell viability assay

Cell viability was evaluated using CCK-8 kits. HK-2 cells were seeded on 96-well plates at a concentration of 5 × 10^4^ cells/well and then cultured for 48 h. The medium was then supplemented with various doses of glucose (0, 10, 20 and 30 mM), and the culture was continued for various durations (0, 12, 24 and 48 h). In addition, HK-2 cells were pretreated with various concentrations of Ori (0, 2.5, 5 and 10 M) for 2 h and then exposed to 30 mM HG for 48 h. Subsequently, CCK-8 reagent (10 μl) was added to each well, and the plate was incubated at 37 °C for 4 h. A microplate reader was then used to measure the absorbance at 540 nm (Molecular Devices, MD, USA).

### Transwell assay

The rate of cell migration was assessed by Transwell assays. Approximately 1 × 10^5^ cells were seeded into the upper chamber of a 24-well plate insert. The lower chamber contained 600 µL of medium supplemented with 10% FBS as a chemical inducer. After one day of incubation, the cells from the top surface of the chambers were removed with cotton swabs. Cells that had penetrated the chamber membranes were fixed with 4% paraformaldehyde at ambient temperature for 20 min and then stained for 5 min with 0.1% crystal violet at the same temperature. A total of 10 random fields (100× magnification) of each insert were examined under a light microscope (Olympus, Japan).

### Immunofluorescence staining

The protein levels of Col-I and FN in HK-2 cells were detected *via* immunofluorescence staining. HK-2 cells were seeded in 24-well dishes at a density of 7 × 10^4^ cells per well, fixed with 4% paraformaldehyde, and permeabilized for 10 min in 0.5% Triton X-100. The cells were subsequently permeabilized with 0.1% Tris-buffered saline (TBS)-Triton X-100 plus 5% bovine serum albumin (BSA) for 1 h at room temperature in the dark. Primary antibodies against Col-I and FN were then added, and the cells were incubated overnight at 4 °C. 4,6-Diamidino-2-phenylindole dihydrochloride (DAPI) staining was performed for 5 min after incubation with the secondary antibody for 1 h. Observations and imaging were conducted under a fluorescence microscope (Olympus, Japan).

### Western blot

Kidney tissues and HK-2 cells were lysed with radioimmunoprecipitation assay (RIPA) buffer (Beijing Solarbio Science and Technology Co., Ltd.) on ice, and the protein concentration was quantified *via* a bicinchoninic acid (BCA) protein assay kit (ASPEN, USA). Equal amounts of protein samples were separated on sodium dodecyl sulfate (SDS)-polyacrylamide minigels and then transferred to polyvinylidene fluoride (PVDF) membranes by electrophoresis in accordance with standard immunoblot procedures. After blocking with 5% nonfat dried milk in Tris-buffered saline (TBS) containing 0.1% Tween-20 (TBST) for 1 h at 37 °C, the membranes were incubated at 4 °C overnight with the following primary antibodies: Wnt4 (1:500, Ab262696, Abcam), glycogen synthase kinase (GSK)-3β (1:500, Ab93926, Abcam), p-GSK-3β (1:500, AP1088, ABclonal), β-catenin (1:1000, Ab223075, Abcam), α-SMA (1:1000, ab7817, Abcam) and β-actin (1:200, BM0627, Boster). The membranes were washed three times with TBST and then incubated with the corresponding secondary antibodies conjugated to horseradish peroxidase for 1 h at room temperature. The protein bands were identified using an enhanced chemiluminescence (ECL) Plus Western blotting Detection System (ImageQuant LAS 4000 mini, USA) and quantified using ImageJ v1.8.0 software (National Institutes of Health).

### qPCR analysis

Total RNA was extracted from kidney tissues and HK-2 cells utilizing TRIzol reagent (Invitrogen, USA) in accordance with the manufacturer’s instructions. A NanoDrop 2000 spectrophotometer (Invitrogen; Thermo Fisher Scientific, Inc.) was used to measure the concentration and purity of the extracted RNA at an absorbance ratio of 260/280 nm. cDNA was subsequently generated *via* reverse transcription using the PrimeScript^TM^ RT reagent kit (Takara Bio, Inc.). Known sequences were used to design specific primers for Wnt4, GSK-3β, β-catenin and α-SMA. Table 1 shows a list of the qPCR primers used. A SYBR® Premix Ex TaqTM II kit (Takara Bio, Inc.) and a CFX96 Touch Sequence Detection system (Bio-Rad Laboratories, Inc., Hercules, CA, USA) were used for the qPCR assay. The following amplification procedure was used: 40 cycles of 95 °C for 1 min, 60 °C for 30 s, and 72 °C for 30 s. The relative expression was evaluated using the 2 − ΔΔCq method.

**Table 1.** Primers used for reverse transcription-quantitative PCR.

Gene	Primers	Size
Wnt4	Forward 5′-AGCCCACAGGGTTTCCA-3′ Reverse 5′-GCTCGCCAGCATGTCTTT-3′	233 bp
GSK-3β	Forward 5′-ATGCCTGTCTCCTCTAACGC-3′ Reverse 5′-GGTCTTGGTGGCGGGTTT-3′	321 bp
β-catenin	Forward 5′-AACGGCTTTCGGTTGAGCTG-3′ Reverse 5′-TGGCGATATCCAAGGGCTTC-3′	147 bp
α-SMA	Forward 5′-ACTGGTATTGTGCTGGACTC-3′ Reverse 5′-TGATGCTGTTATAGGTGGTT-3′	403 bp
β-actin	Forward 5′-TCAGGTCATCACTATCGGCAAT-3′ Reverse 5′-AAAGAAAGGGTGTAAAACGCA-3′	432 bp

### Statistical analysis

All the data are presented as the means ± standard deviations (SDs). The means of the various groups were compared by one-way or two-way analysis of variance or an unpaired Student’s t test. A value of *p* < 0.05 indicated a significant difference. The statistical analyses were conducted using SPSS 21.0 and GraphPad Prism 8.

## Results

### Effects of Ori on general changes and biochemical indices in diabetic rats

**Figure 2.**
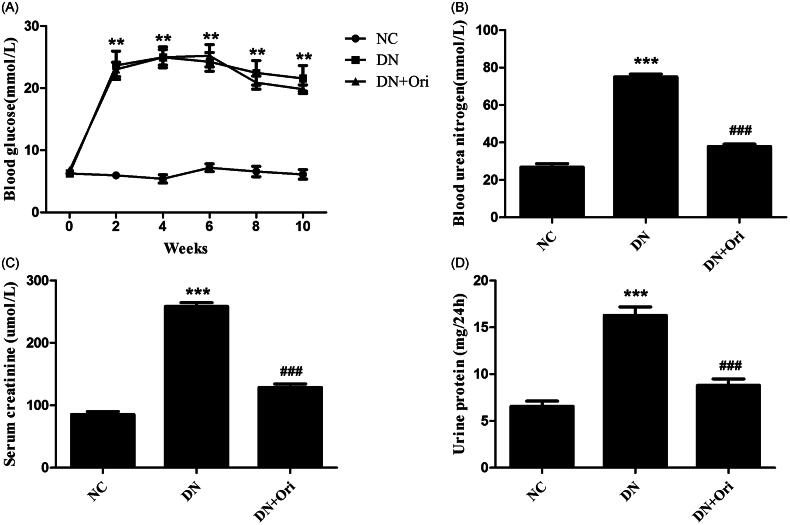
Effects of Ori on general changes and biochemical indices in diabetic rats. (A) Effect of Ori on blood glucose. (B–D) Effect of Ori on BUN, Scr and 24-h urinary protein concentrations. **p < 0.01 vs. the NC group; ***p < 0.001 vs. the NC group; ^###^*p* < 0.001 vs. the DN group. Ori: oridonin; BUN: blood urea nitrogen; Scr: serum creatinine; NC: normal control; DN: diabetic nephropathy; DN + Ori: diabetic nephropathy + oridonin.

The blood glucose levels of the DN group were markedly greater than those of the NC group; however, there was no significant difference in blood glucose levels between the DN and DN + Ori groups (Figure 2A). Compared with those in the NC group, the BUN, Scr and 24-h urine albumin levels in the DN group were significantly greater (Figure 2B–D). Amazingly, the BUN, Scr and 24-h urine albumin levels were significantly lower in the DN + Ori group than in the DN group after treatment with Ori, as shown in Figure 2B–D. These data suggested that Ori could alleviate renal injury independent of blood glucose levels in diabetic rats.

### Effects of Ori on renal pathological changes in diabetic rats

**Figure 3.**
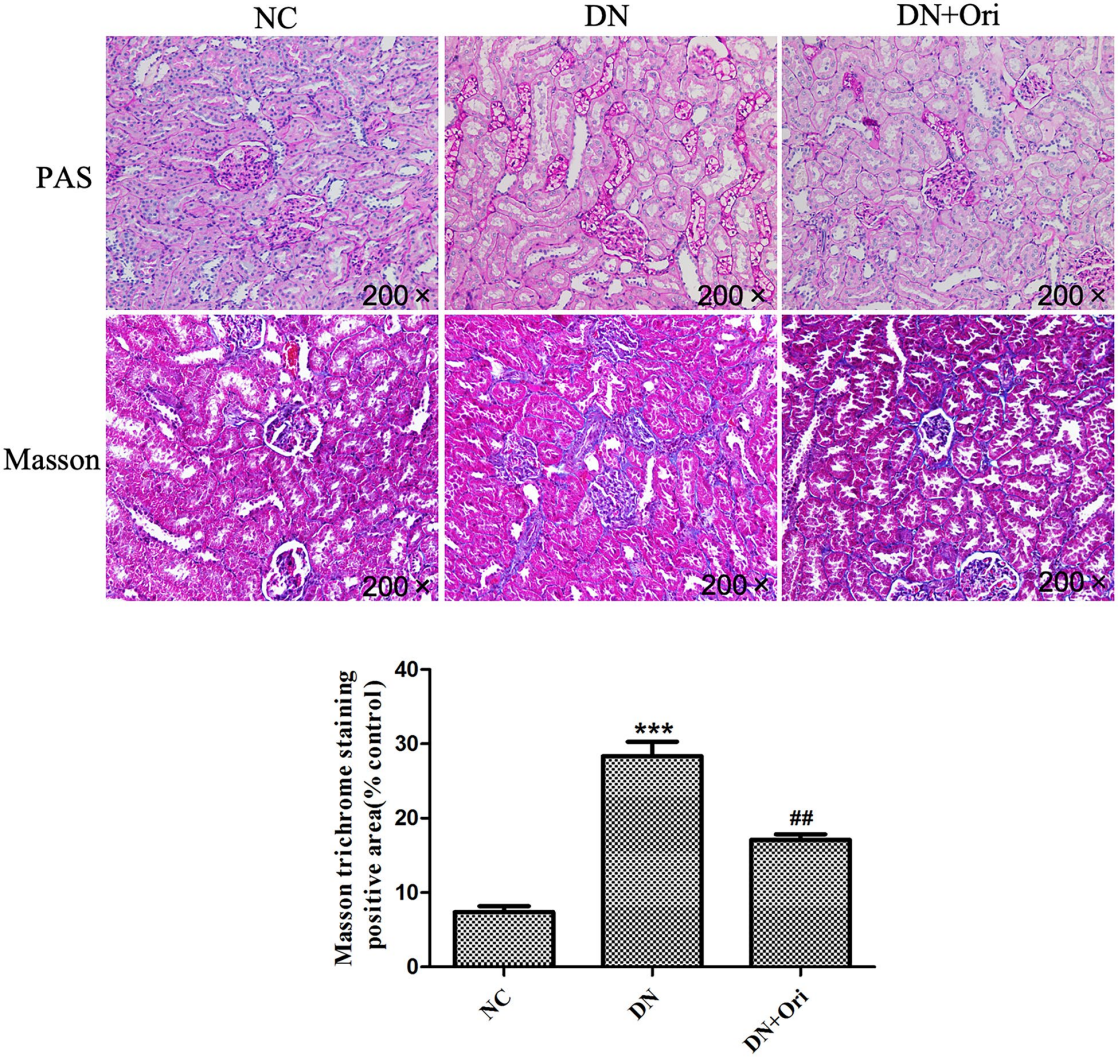
Effects of Ori on renal pathological changes in diabetic rats. PAS and MT staining of kidney tissues. **p < 0.01 vs. the NC group, ^###^*p* < 0.001 vs. the DN group. PAS: periodic acid-Schiff; MT: Masson’s trichrome; NC: normal control; DN: diabetic nephropathy; DN + Ori: diabetic nephropathy + oridonin.

The renal pathological changes in the different groups of rats are shown in Figure 3. PAS staining revealed clear glomerular and tubular structures in the NC group, with a small amount of purple–red PAS-positive staining material visible and no obvious abnormalities observed. In the DN group, the mesangial matrix was slightly hyperplastic, with an increase in PAS-positive staining compared to that in the NC group. However, compared with those in the DN group, the number of PAS-positive substances was significantly lower in the DN + Ori group (Figure 3). Similarly, Masson staining revealed that significantly more collagen fibers were deposited in the interstitial region of the glomeruli and tubules, namely, in the blue bundle-like substance in the interstitial area of the glomeruli and tubules, than in the NC group. After Ori treatment, the DN + Ori group had significantly lower levels of blue bundle-like substances within the entire field of view than did the DN group (Figure 3).

### Effects of Ori on renal fibrosis in diabetic rats

**Figure 4.**
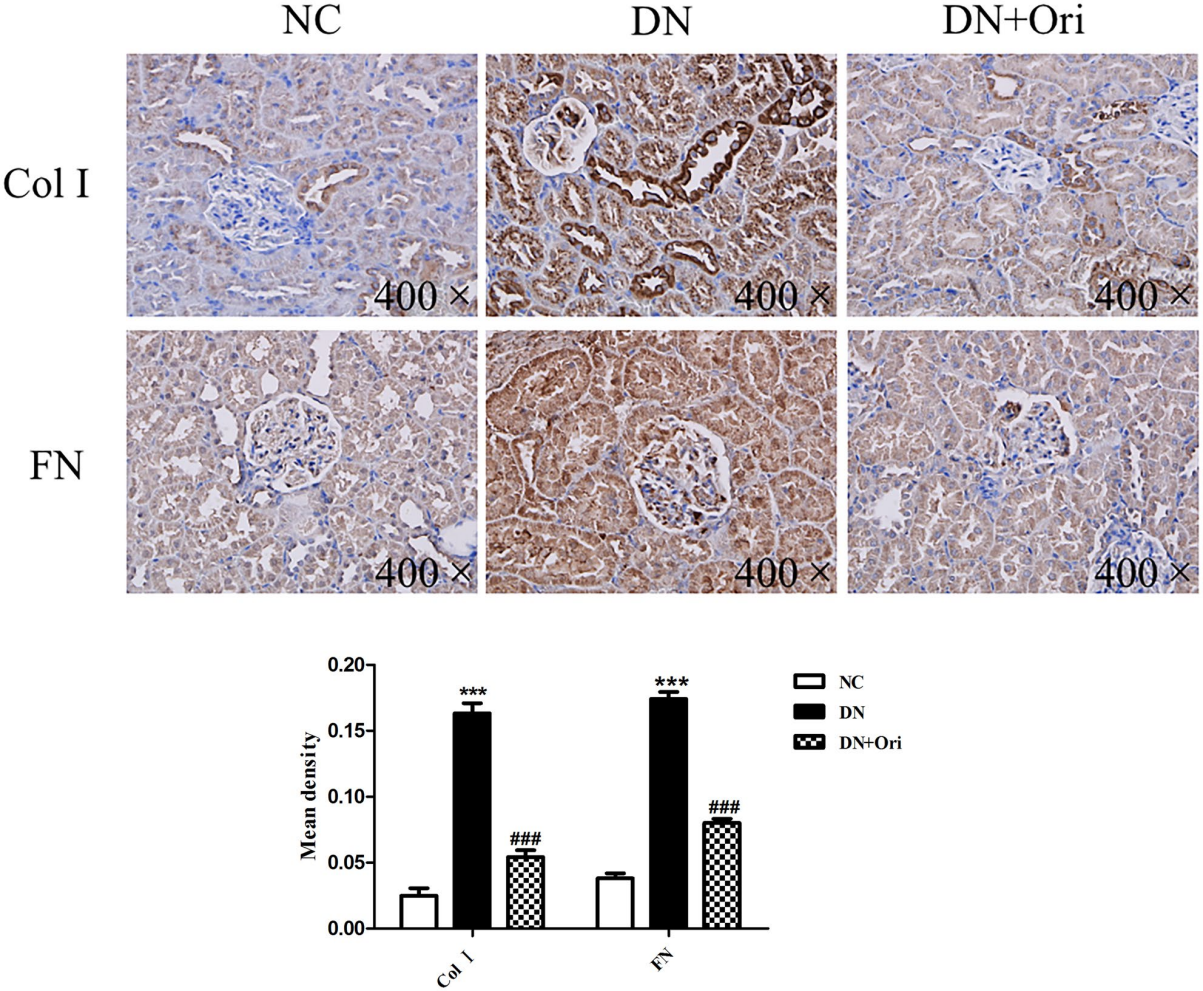
Effects of Ori on renal fibrosis in diabetic rats. Immunohistochemical analysis of Col I and FN expression in kidney tissues. ***p < 0.001 vs. the NC group, ^###^*p* < 0.001 vs. the DN group. Col I, collagen I; FN: fibronectin; NC: normal control; DN: diabetic nephropathy; DN + Ori: diabetic nephropathy + oridonin.

Renal fibrosis was evaluated to further verify the influence of Ori on antifibrotic activity. Immunohistochemical analysis of Col I and FN, which are markers of renal fibrosis, was clearly greater in the DN group than in the NC group; however, compared with those in the DN group, the expression of Col I and FN was significantly lower in the Ori treatment groups (Figure 4). Our findings indicated that Ori reduces renal fibrosis in diabetic model rats.

### Expression of the Wnt/β-catenin signaling pathway in the kidneys of diabetic rats

**Figure 5.**
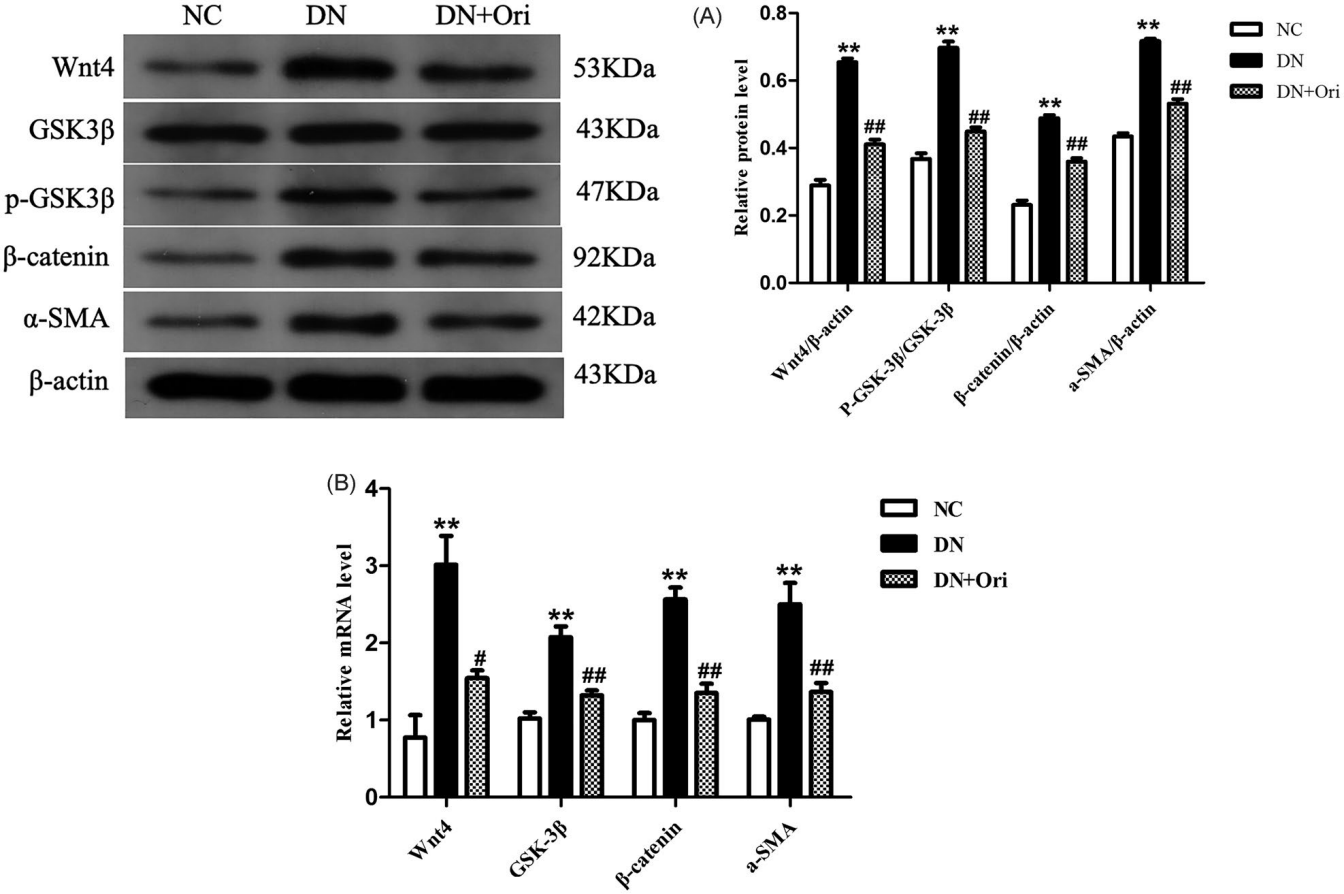
Expression of the Wnt/β-catenin signaling pathway in the kidneys of diabetic rats. (A) Protein expression levels of Wnt4, GSK3β, p-GSK3β, β-catenin and α-SMA in rat kidney tissues. (B) qPCR analysis of the mRNA expression of Wnt4, GSK3β, β-catenin and α-SMA in rat kidney tissues. **p < 0.01 vs. the NC group; ^#^*p* < 0.05 vs. the DN group; ^##^*p* < 0.01 vs. the DN group; ^###^*p* < 0.001 vs. the DN group. p-GSK3β: phospho-glycogen synthase kinase-3β; α-SMA: α-smooth muscle actin; qPCR: quantitative polymerase chain reaction.

Next, we evaluated the expression of critical molecules at both the mRNA and protein levels to assess the impact of Ori on the Wnt/β-catenin signaling pathway, which has been shown to play important roles in the progression of diabetic renal fibrosis. The results of the Western blot analysis revealed that the protein expression levels of Wnt4, p-GSK3β, β-catenin, and α-SMA in the kidney tissue of diabetic rats were considerably greater than those in the corresponding control groups. After Ori treatment, the protein expression of Wnt4, p-GSK3β, β-catenin, and α-SMA was significantly lower than that in the DN group (Figure 5A). The results of the qPCR were consistent with those of the Western blot analysis, and treatment with Ori significantly decreased the Wnt4, GSK3β, β-catenin and α-SMA mRNA expression levels in the kidney tissue of diabetic rats (Figure 5B).

### Effects of Ori on cell viability

**Figure 6.**
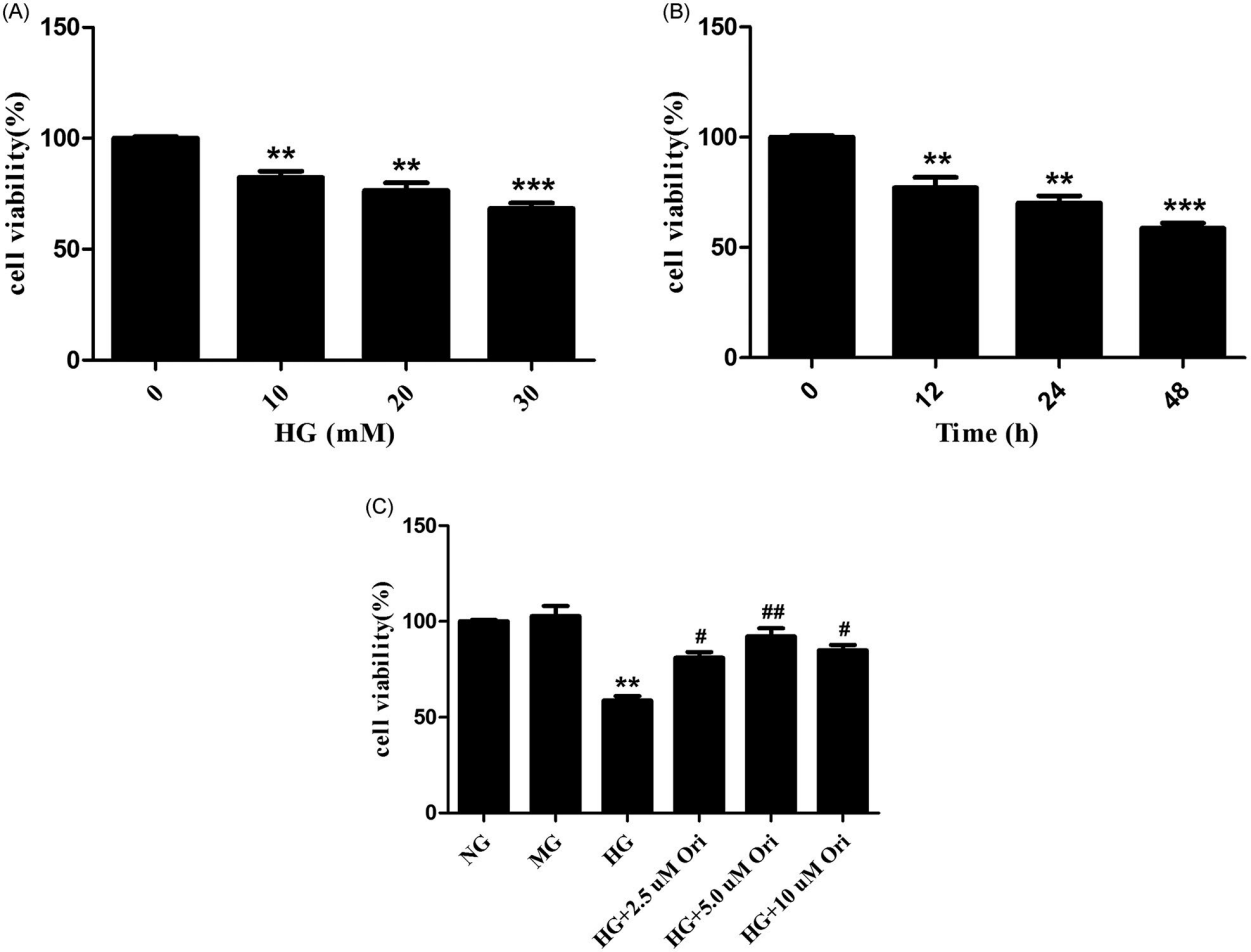
Effects of Ori on cell viability. (A) HK-2 cells were treated with different concentrations of HG (0, 10, 20, or 30 mM) for 48 h, after which their viability was detected via CCK-8 assays. (B) HK-2 cells were treated with HG (30 mM) for different durations (0, 12, 24, and 48 h), and their viability was detected by CCK-8 assays. (C) HK-2 cells were pretreated with different concentrations of Ori (2.5, 5, or 10 μM) for 2 h and then treated with 30 mM HG for 48 h, after which their viability was detected via CCK-8 assays. **p < 0.01 vs. the NG group; ***p < 0.001 vs. the NG group; ^#^*p* < 0.05 vs. the HG group; ^##^*p* < 0.01 vs. the HG group; HG: high glucose; CCK-8: Cell Counting Kit-8; NG: normal glucose control (5.5 mmol/L glucose); MG: mannitol control (19.5 mmol/L mannitol + 5.5 mmol/L glucose); NG + Ori: 5.5 mmol/L glucose + 5 μmol/L oridonin; HG: 30.0 mmol/L glucose; HG + Ori: 30.0 mmol/L glucose + 5 μmol/L oridonin.

HK-2 cell viability was evaluated by CCK-8 analysis. The results showed that high glucose (HG) decreased the viability of HK-2 cells in a time- and dose-dependent manner (Figure 6A,B). Therefore, in the following experiments, HK-2 cells were treated with 30 mM HG for 48 h. As shown in Figure 6C, the viability of HK-2 cells was lower in the HG-treated group than in the normal glucose control (NG) and mannitol control (MG) groups. However, treatment with Ori (HG + Ori) reversed these changes, and the best protective effect was observed with an Ori concentration of 5 mM. Hence, 5 mM was selected as the optimal Ori concentration for the *in vitro* experiment.

### Ori inhibited fibrosis in HG-treated HK-2 cells

**Figure 7.**
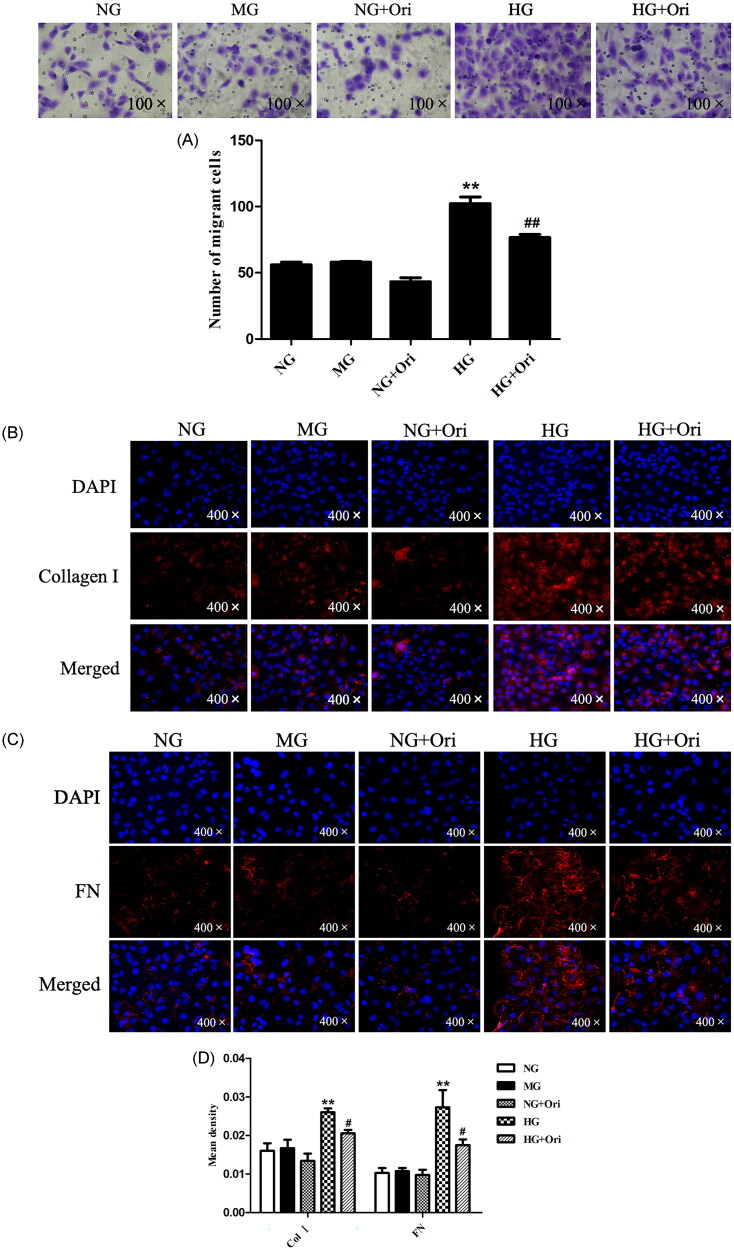
Ori inhibited fibrosis in HG-treated HK-2 cells. (A) Transwell assays were used for the assessment of cell migration. (B & C) Col I and FN were detected by specific immunofluorescence staining. (D) Quantification of the immunofluorescence staining of Col I and FN. **p < 0.01 vs. the NG group; ^#^*p* < 0.05 vs. the HG group; ^##^*p* < 0.01 vs. the HG group; Col I: collagen I; FN: fibronectin; HG: high glucose; NG: normal glucose control (5.5 mmol/L glucose); MG: mannitol control (19.5 mmol/L mannitol + 5.5 mmol/L glucose); NG + Ori: 5.5 mmol/L glucose + 5 μmol/L oridonin; HG: 30.0 mmol/L glucose; HG + Ori: 30.0 mmol/L glucose + 5 μmol/L oridonin.

First, the impact of Ori on cell migration was examined using a transwell assay. We found that the migration of HK-2 cells increased under hyperglycemic conditions, which indicated the destruction of epithelial layers and the loss of cell–cell adhesion compared with those in the NG and MG groups. However, Ori treatment decreased HG-induced cell migration (Figures 7A). Thus, even in the environment of diabetes, Ori may prevent renal tubular cell dysfunction and preserve the barrier function of the renal tubular epithelial layer. Second, we evaluated the expression of fibrosis-related indicators by immunofluorescence analysis. As shown in Figure 7B–D, Col I and FN expression in HK-2 cells was significantly increased after exposure to HG. However, compared with the HG treatment, the Ori treatment significantly decreased the Col I and FN expression levels; these results were consistent with those of the *in vivo* experiments. The protein levels did not significantly differ between the control groups (NG, MG, NG + Ori) (Figure 7B–D).

### Expression of proteins in the wnt/β-catenin signaling pathway in HG-treated HK-2 cells

**Figure 8.**
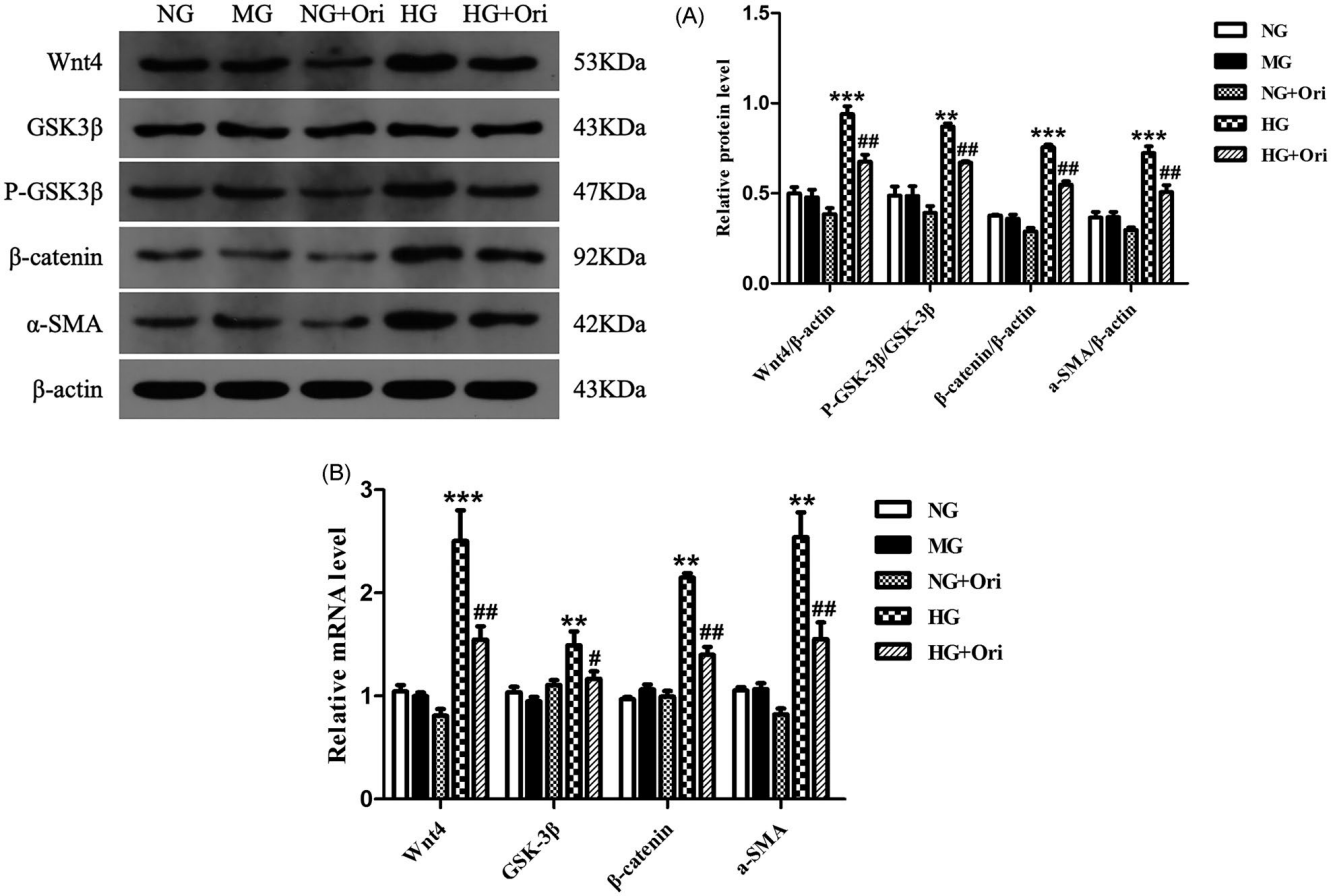
Expression of proteins in the Wnt/β-catenin signaling pathway in HG-treated HK-2 cells. (A) Protein expression levels of Wnt4, GSK3β, p-GSK3β, β-catenin and α-SMA in HK-2 cells. (B) qPCR analysis of the mRNA expression of Wnt4, GSK3β, β-catenin and α-SMA in HK-2 cells. **p < 0.01 vs. the NC or NG group, ***p < 0.001 vs. the NC or NG group, ^#^*p* < 0.05 vs. the DN or HG group, ^##^*p* < 0.01 vs. the DN or HG group, and ^###^*p* < 0.001 vs. the DN or HG group. p-GSK3β, phospho-glycogen synthase kinase-3β; α-SMA: α-smooth muscle actin; qPCR: quantitative polymerase chain reaction.

Immunoblotting revealed that the protein expression levels of Wnt4, p-GSK3β, β-catenin, and α-SMA in HK-2 cells treated with HG were considerably greater than those in the corresponding control cells. Ori treatment significantly decreased Wnt4, p-GSK3β, β-catenin and α-SMA protein expression levels (Figure 8A). According to the qPCR results, treatment with HG considerably increased the Wnt4, GSK3β, β-catenin, and α-SMA mRNA expression levels in HK-2 cells compared with those in the healthy control group. However, Ori treatment significantly decreased the increase in Wnt4, GSK3β, β-catenin and α-SMA mRNA expression induced by HG (Figure 8B).

### Ori attenuated renal fibrosis by inhibiting the wnt/β-catenin signaling pathway

To explore the specific mechanism through which Ori attenuates renal fibrosis in DN, we knocked down β-catenin in HK-2 cells using β-catenin siRNA under high-glucose conditions. We also detected the cell migration and the expression levels of Wnt/β-catenin signaling effectors in each group. Transwell assays showed that β-catenin downregulation and Ori treatment had similar effects on decreasing cell migration induced by hyperglycemia (Figure 9A). In addition, immunofluorescence analysis revealed that β-catenin downregulation markedly inhibited the expression of Col I and FN, which was consistent with the results of Ori treatment (Figure 9B–D). Similarly, according to the immunoblotting and qPCR results, both the protein and mRNA levels of Wnt4, p-GSK3β, β-catenin, and α-SMA were significantly suppressed by Ori treatment and β-catenin downregulation (Figure 9E–F). On the other hand, β-catenin was overexpressed by plasmid transfection, and the overexpression of β-catenin increased HG-induced cell migration and cell fibrosis (Figure 9A–F). These findings collectively demonstrated that Ori inhibits the Wnt/β-catenin signaling pathway to ameliorate kidney fibrosis.

Figure 9.Ori attenuated renal fibrosis by inhibiting the Wnt/β-catenin signaling pathway.(A) Transwell assays were used for the assessment of cell migration. (B & C) Detection of Col I and FN expression via immunofluorescence staining. (D) Quantification of the immunofluorescence staining of Col I and FN. (E) Protein expression levels of Wnt4, GSK3β, p-GSK3β, β-catenin and α-SMA in HK-2 cells. (F) qPCR analysis of the mRNA expression of Wnt4, GSK3β, β-catenin and α-SMA in HK-2 cells. The results are representative of three independent experiments. ^#^*p* < 0.05 vs. the HG group, ^##^*p* < 0.01 vs. the HG group, ^###^*p* < 0.001 vs. the HG group. KD: knockdown; OE: overexpression.

**Figure:**
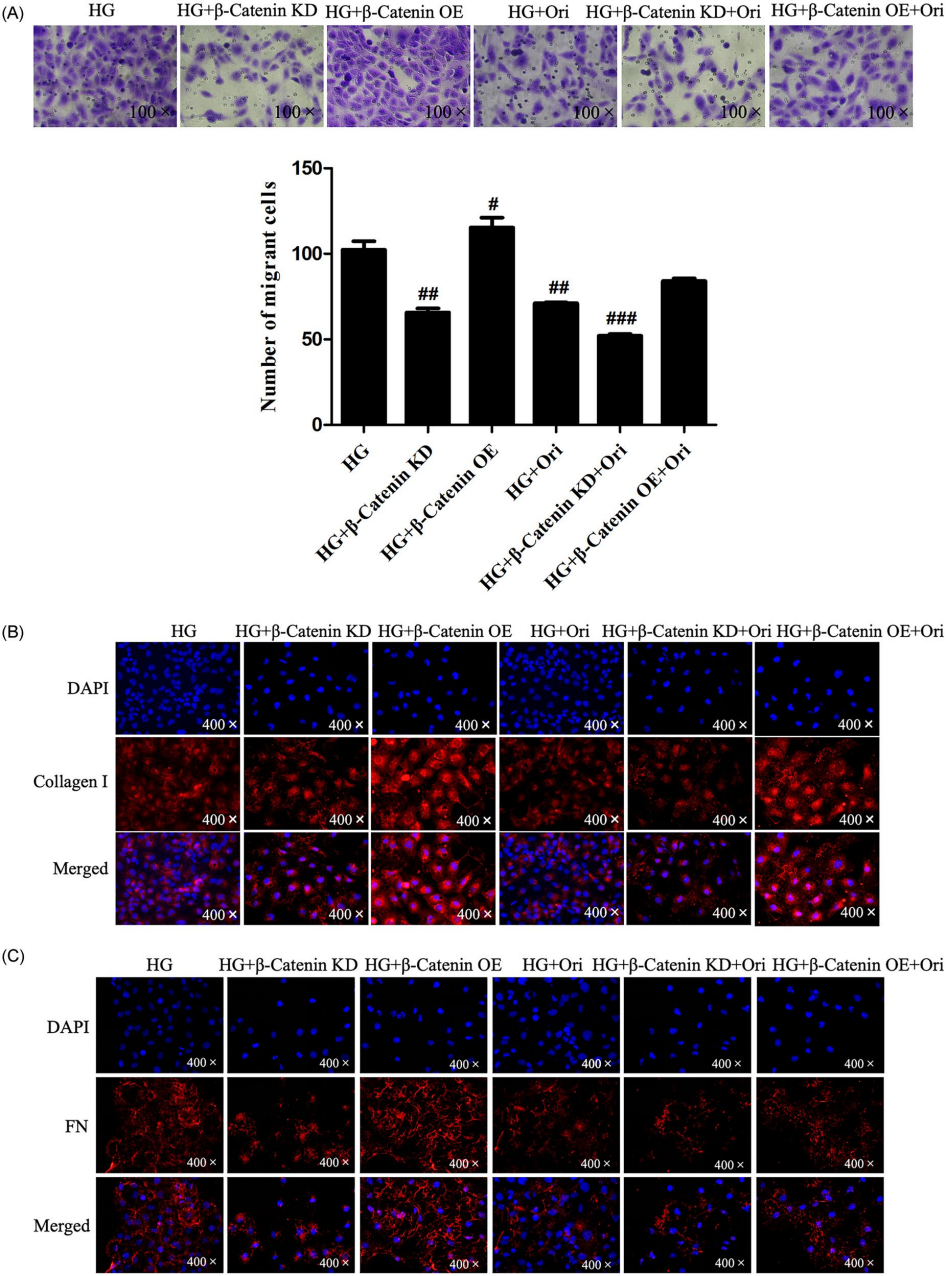

**Figure:**
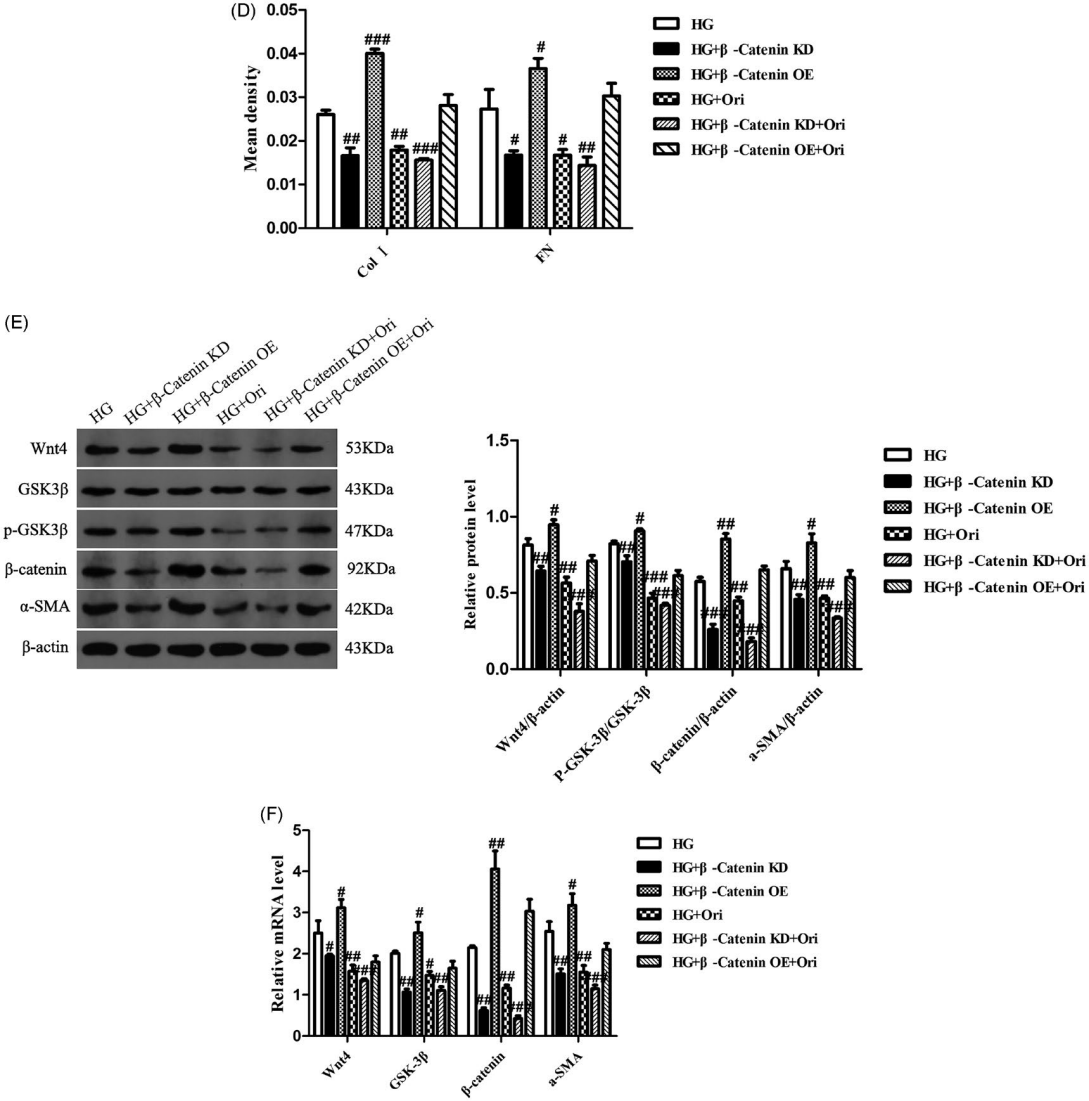

## Discussion

DN is a serious microvascular complication of diabetes and the leading cause of CKD and ESRD worldwide [27,28]. However, the etiology of DN is not clearly understood. In hyperglycemia renal TEC damage occurs earlier than damage in the glomerulus. It has been established that partial epithelial–mesenchymal transition (EMT) is a crucial step in the development of fibrosis [29]. Tubulointerstitial fibrosis is a progressive and irreversible process that has a significant impact on the occurrence and progression of DN [5,6].

The Wnt/β-catenin signaling pathway, which was identified in 1982, drives tissue organization during embryogenesis and maintains the structure of organs and tissues throughout life [30,31]. By inducing interactions between the receptor Frizzled (FZD) and lipoprotein receptor-related protein 5 (LRP5) or LRP6, Wnt ligands initiate β-catenin-dependent signaling, which is also called canonical Wnt signaling. Subsequently, β-catenin, which is known as a transcription factor, accumulates and translocates into the nucleus, where it can form a complex with T-cell factor (TCF) and lymphoid enhancer factor (LEF) to trigger Wnt-dependent gene expression [13]. Sustained activation of Wnt/β-catenin signaling can promote kidney fibrosis in CKD patients, podocyte destruction, proteinuria, and long-lasting tissue damage following acute renal injury (AKI) [13]. Our current study revealed that diabetic rat kidney tissue and HG-treated HK-2 cells had considerably greater mRNA and protein expression levels of important molecules involved in Wnt/β-catenin signaling, such as Wnt4, p-GSK-3β and β-catenin, than did the normal control cells. These findings demonstrated that Wnt/β-catenin signaling was activated in DN. Ren and colleagues observed that the expression levels of Wnt4 and β-catenin were clearly increased in diabetic rats and HG-stimulated rat kidney tubular epithelial cells (NRK-52E) [32]. He et al. [33] demonstrated that HK-2 cells treated with HG had markedly greater Wnt4 and β-catenin expression levels than did untreated control cells. In addition, researchers have shown that β-catenin expression in kidney tissues from diabetic patients is significantly greater than that in tissues from nondiabetic patients, as revealed by immunohistochemistry. Zhang et al. [34] reported that the mRNA and protein expression levels of Wnt4, β-catenin and p-GSK-3β in tissues from DN patients were significantly elevated compared with those in normal renal tissues. We obtained similar results to those of these reports.

An increasing number of studies have shown that modulating the Wnt/β-catenin signaling pathway with specific inhibitors or natural compounds or by modulating gene expression can alleviate tubular EMT and interstitial fibrogenesis in TECs incubated under HG conditions and in animal models of DN. Ren et al. [32] confirmed that XAV939, an inhibitor of Wnt/β-catenin, can inhibit the elevated expression of Wnt4, β-catenin, α-SMA, FN and transforming growth factor-β (TGF-β) and restore E-cadherin activity in HG-treated NRK-52E cells. Wang et al. [35] reported that astragaloside IV inhibits increases in the protein expression levels of N-cadherin, α-SMA, Wnt1 and β-catenin in DN rats. Treatment with astragaloside IV and XAV939 reduces the levels of β-catenin and p-GSK3β in HK-2 cells treated with HG and blocks the nuclear translocation of β-catenin. Furthermore, astragaloside IV therapy enhances E-cadherin synthesis while decreasing the synthesis of α-SMA, vimentin, N-cadherin, FN and Col IV. Xiang et al. [36] reported that *Salvia miltiorrhiza* extracts decrease the expression levels of Wnt4, β-catenin and TGF-β1 and alleviate tubulointerstitial fibrosis in kidney tissues from diabetic rats. Dai et al. [37] reported that total glycosides from Rehmannia glutinosa leaves downregulate Wnt4, β-catenin and TGF-β1 expression and decrease the renal tubular injury score in a rat model of DN. Zhu et al. [38] demonstrated that the expression of dedicator of cytokinesis 4 (DOCK4), a guanine nucleotide exchange factor (GEF) that can induce the Wnt/β-catenin signaling pathway, is increased in HG-stimulated HK-2 cells and kidney tissues from diabetic mice. In HK-2 cells, the knockdown of DOCK4 clearly decreased the expression of β-catenin and α-SMA and significantly increased that of E-cadherin. In this study, we discovered that the administration of Ori suppressed the Wnt/β-catenin signaling pathway, as revealed by significant reductions in Wnt4, p-GSK3β and β-catenin expression and alleviated fibrosis, as revealed by marked reductions in α-SMA, Col I and FN expression *in vitro* and *in vivo*. These results were consistent with previous reports.

Ori is a diterpenoid chemical derived from traditional Chinese medicinal plants that has many biological effects, including antitumour, anti-inflammatory, antifibrotic, immunoregulatory and antioxidant effects [16–21]. Our previous study revealed the anti-inflammatory effects of Ori in diabetic rats [25]. The goal of this study was to determine whether Ori exerts antifibrotic effects on DN by blocking the Wnt/β-catenin signaling pathway. It has been reported that Ori suppresses cancer cell proliferation and migration by preventing Wnt/β-catenin signal transduction [17, 39–41]. Our current study demonstrated that Ori reduces renal fibrosis in DN by regulating the Wnt/β-catenin signaling pathway. A few natural compounds have been demonstrated to protect against renal fibrosis by modulating Wnt/β-catenin signaling [42–44]. The antifibrotic effects of Ori in DN make this natural compound a novel therapeutic target in DN.

In conclusion, our study provides the first line of evidence showing that Ori alleviates fibrosis in DN by inhibiting the Wnt/β-catenin signaling pathway both *in vitro* and *in vivo*. These experimental results indicate that Ori could be a novel therapeutic target in DN in the future.

## Data Availability

The data that support the findings of this study are available from the corresponding author upon reasonable request.
